# HIV-infected mental health patients: characteristics and comparison with HIV-infected patients from the general population and non-infected mental health patients

**DOI:** 10.1186/1471-244X-13-35

**Published:** 2013-01-23

**Authors:** Annemiek Schadé, Gerard van Grootheest, Johannes H Smit

**Affiliations:** 1 Expert and Treatment Center on HIV and Mental Health, GGZ inGeest, VU University Medical Center, AJ Ernststraat 1187, Amsterdam, 1081 HL, The Netherlands

**Keywords:** HIV, Mental health, Depression, Homosexual men, Drugs, Prevention

## Abstract

**Objectives:**

HIV-infected patients are at increased risk of developing mental health symptoms, which negatively influence the treatment of the HIV-infection. Mental health problems in HIV-infected patients may affect public health. Psychopathology, including depression and substance abuse, can increase hazardous sexual behaviour and, with it, the chance of spreading HIV. Therefore, it is important to develop an optimal treatment plan for HIV-infected patients with mental health problems. The majority of HIV-infected patients in the Netherlands (almost 60%) are homosexual men.

The main objectives of this study were to describe the clinical and demographic characteristics of patients with HIV who seek treatment for their mental health symptoms in the Netherlands. Secondly, we tested whether HIV infected and non-infected homosexual patients with a lifetime depressive disorder differed on several mental health symptoms.

**Methods:**

We compared a cohort of 196 patients who visited the outpatient clinic for HIV and Mental Health with HIV-infected patients in the general population in Amsterdam (ATHENA-study) and with non-HIV infected mental health patients (NESDA-study). DSM-IV diagnoses were determined, and several self-report questionnaires were used to assess mental health symptoms.

**Results:**

Depressive disorders were the most commonly occurring diagnoses in the cohort and frequent drug use was common. HIV-infected homosexual men with a depressive disorder showed no difference in depressive symptoms or sleep disturbance, compared with non-infected depressive men. However, HIV-positive patients did express more symptoms like fear, anger and guilt. Although they showed significantly more suicidal ideation, suicide attempts were not more prevalent among HIV-infected patients. Finally, the HIV-infected depressive patients displayed a considerably higher level of drug use than the HIV-negative group.

**Conclusion:**

Habitual drug use is a risk factor for spreading HIV. It is also more often diagnosed in HIV-infected homosexual men with a lifetime depression or dysthymic disorder than in the non-infected population. Untreated mental health problems, such as depressive symptoms and use of drugs can have serious repercussions. Therefore, general practitioners and internists should be trained to recognize mental health problems in HIV-infected patients.

## Background

HIV is a highly stigmatized, chronic disease with a substantial co-occurrence of mental health problems [1]. On the one hand, patients with mental health problems are at increased risk of contracting HIV. On the other hand, HIV-infected patients are at increased risk of developing mental health problems compared with the general population [2]. Mental health problems in HIV-infected patients have a negative influence on the treatment, adherence to treatment, and prognosis of the HIV-infection [3,4]. Similar effects have been determined for other chronic diseases, such as diabetes and chronic obstructive pulmonary disease (COPD) [5,6]. However, there are some important differences between HIV and other chronic diseases. First, HIV-infected patients suffer more from shame, stigma, and discrimination, which can also cause mental health problems [7,8]. Second, mental health problems in HIV-infected patients can affect public health. Psychopathology like depression and substance abuse can increase hazardous sexual behaviour and, with it, the chance of spreading HIV [9,10].

The most common mental disorders among HIV-infected patients are depression and depressive symptoms. The chance of developing a depressive disorder is two times higher in HIV-infected patients than in HIV-negative comparison subjects [11]. Depressive symptoms are even more common: 54% of HIV-infected patients reported depressive symptoms in the past week [12]. Although it has a high prevalence, only fewer than 50% of the cases are recognized clinically [13]. Apart from the HIV-infection, stigma and discrimination are associated with an increase in depressive symptoms among HIV-infected patients [14]. In addition, the risk of suicide and suicide attempts is higher among HIV-infected patients compared with the general population and compared with patients with other chronic diseases [15].

Conversely, several mental health problems have been identified as risk factors for contracting HIV [16,17]. For example, depressive symptoms and the use of alcohol or drugs, such as amphetamine, cannabis and cocaine, are associated with increased sexually risky behaviour and an increased risk of contracting HIV, particularly among men who have sex with men(MSM) [18-20]. Certain personality traits of the Five-Factor Module of Personality [21] are also associated with an increased risk. High scores on neuroticism (chronic emotional distress, e.g. anxiety, depression, impulsiveness) and low scores on agreeableness (altruism, modesty, trust) and conscientiousness are associated with HIV risk behaviours [22].

Mental health problems can arise and increase during different stages of HIV, such as for example, immediately after testing HIV-positive or after many years of living with HIV [23]. Despite the fact that mental health problems are poorly recognized by clinicians [13], some of the HIV-infected patients with mental health problems will seek help at an institution for mental health. To develop an optimal treatment plan for this group of patients, it is important to determine the demographic characteristics, psychiatric diagnoses and severity of the psychopathology.

In the Netherlands there is a database available that includes demographic data of almost all HIV-positive patients (ATHENA) [24]. We are interested in knowing whether the mental health treatment seeking HIV-infected population in Amsterdam differs from the general HIV-infected population in Amsterdam on demographic and medical characteristics.

The majority of HIV-infected patients in the Netherlands are homosexual men. In 2010, 7532 men (58%) of the infected HIV population (13.035) were men who have sex with men (MSM) [24]. It is interesting, therefore, to compare them with homosexual HIV-negative men.

It is unknown whether the presence of HIV has an effect on the severity of psychiatric symptoms of patients with mental health problems. We hypothesize that HIV may cause more severe symptoms and is associated with more co-morbid substance use among patients with a depressive or dysthymic disorder. Furthermore, HIV-infected patients with mental health problems probably show a different pattern in personality traits like neuroticism, agreeableness and conscientiousness, compared with non-infected patients with the same mental health problems.

If treatable mental health problems, such as depressive symptoms, alcohol and drug abuse and, to a lesser extent, personality traits are recognised and assessed, the prognosis of the HIV-infection might be improved and the risk of spreading HIV diminished. The present study had two main objectives. First, we described the clinical and demographic characteristics of patients with HIV who seek treatment for their mental health problems in a specialized outpatient clinic for HIV and mental health. We subsequently compared these patients with the general population of HIV-positive patients in the Netherlands. Second, we tested whether HIV-infected homosexual patients and non-infected homosexual patients with a lifetime depressive or dysthymic disorder differed on 1) depressive symptoms, 2) co-morbid use of alcohol and drugs, and 3) personality traits.

The chance of developing psychopathology is higher in HIV-infected patients than in HIV-negative comparison subjects [2]. However, we also expect that these symptoms are more severe, due to the influence of a lifelong disease, exacerbated by stigma and discrimination.

## Method

### HIV-infected patients with mental health problems

We conducted the study at the outpatient clinic for HIV and Mental Health at GGZ inGeest in Amsterdam, the Netherlands, between March 2006 and September 2009. This clinic was founded in 1984 and specializes in the assessment and treatment of mental health problems in HIV-infected patients. Patients can be referred to the clinic by their infectious disease specialist, HIV-nurse, family doctor, or regular psychologist or psychiatrist.

New patients visiting the outpatient clinic were asked to participate in this study during their first appointment. The design and the purpose of the study were explained and the patients gave written informed consent. The Medical Ethics Review Committee of the VU University Medical Center approved the research protocol and gave permission for publication.

At the intake, trained staff members obtained a DSM-IV diagnosis by assessment, using the M.I.N.I. International Neuropsychiatric Interview (M.I.N.I. Plus) [25]. Demographic data were recorded and all participants were asked to fill in the assessment lists. The assistant-coordinator of the research program contacted the patients if the assessments lists were not returned. All eligible patients with HIV and mental health problems who gave informed consent and completed the questionnaires were included in the cohort (N = 196).

### Control subjects: the general HIV-population of Amsterdam

To compare demographic and clinical characteristics of the cohort of patients from the outpatient clinic for HIV and Mental Health in Amsterdam with the general HIV-positive population of Amsterdam, we selected HIV infected patients from the ATHENA national observational HIV cohort in the Netherlands. ATHENA collects anonymous data obtained during routine clinical care from all HIV-infected patients in the Netherlands who receive care at one of the 25 designated HIV treatment centres [26]. In 2007, the total HIV-infected population collected by ATHENA consisted of 12,570 patients. For the comparison with patients from the outpatient clinic for HIV and Mental Health, we used 5304 HIV-infected patients from Amsterdam.

### Control subjects: homosexual men with a lifetime dysthymic or depressive disorder

For the comparison between HIV infected homosexual patients and non-infected homosexual patients, we selected homosexual men with a lifetime dysthymic or depressive disorder from both the original HIV research cohort (N = 84) and the Netherlands Study of Depression and Anxiety (NESDA) (N = 60). NESDA is a longitudinal cohort study that investigates the long-term course of depressive and anxiety disorders. A description of the rationales, methods, and recruitment strategy is reported elsewhere [27]. In this study, DSM-IV diagnoses were obtained using the World Health Organization Composite International Diagnostic Interview (CIDI V 2.1). Although a different diagnostic instrument was used in NESDA than at the intake at the clinic for HIV and mental health, both the CIDI and the MINI assess psychiatric diagnoses according to the DSM-IV classification. Previous studies have shown that diagnoses obtained with the MINI and CIDI were comparable. In the comparison between the MINI and the CIDI the Kappa values were greater than 0.5, except for simple phobia and GAD. Sensitivity was 0.7 or greater for all but four values (panic, agoraphobia, simple phobia, and lifetime bulimia). Specificity was 0.7 or greater for all diagnoses [28].

Demographics were assessed and patients completed several questionnaires. All respondents signed an informed consent at baseline assessment. A total of 2981 respondents, aged 18–65, were recruited from the general population (19%), primary care (54%) and mental health care organizations (27%) to represent various settings and stages of psychopathology. Selection of homosexual men with a lifetime depressive or dysthymic disorder from the NESDA database yielded a total group of 60 patients without HIV.

### Measures

The self-report questionnaire was used to obtain measures in three domains: severity of symptoms, personality traits, and substance use. The specific instruments were all part of the NESDA study and allowed us to compare the HIV study participants with the NESDA study participants and included the following assessment lists:

Inventory of Depressive symptoms (IDS) [29]: the 30 item Inventory of Depressive Symptomatology (IDS) is designed to assess the severity of depressive symptoms.

Beck Anxiety Index (BAI) [30]: the Beck Anxiety Inventory (BAI) is a 21-item self-report instrument that assesses the overall severity of anxiety. The BAI was developed to address the need for an instrument that would reliably discriminate anxiety from depression while displaying convergent validity.

Alcohol Use Disorders Identification Test (AUDIT) [31]: the AUDIT identifies persons with hazardous and harmful patterns of alcohol consumption.

NEO Five-Factor Inventory (NEO-FFI) [21]: personality traits of the Five-Factor Module of Personality, Openness (Aesthetic Interests, Intellectual Interests, Unconventionality); Agreeableness (Nonantagonistic Orientation, Prosocial, Orientation); Conscientiousness (Orderliness, Goal-Striving, Dependability); Extraversion (Positive Affect, Sociability, Activity); and Neuroticism (Negative Affect, Self- Reproach).

Fear Questionnaire [32]: the Fear Questionnaire (FQ) is part of a standard brief self-rated form. The complete form also includes one specific main target phobia, a global phobia rating, and five associated anxiety and depression symptoms.

Mood and Anxiety Symptom Questionnaire (MASQ) [33]: the model is based on the assumption that mood can be dissected into two components, negative affect (NA) and positive affect (PA) and a third dimension of somatic arousal (SA). Whereas NA is characterized by aversive emotional states, such as fear, anger, and guilt that are associated with both anxiety and depression, PA represents positive emotional states such as feeling active, excited, delighted, enthusiastic, and interested. A lack of PA is described as feeling ‘tired and sluggish’ and is associated with depressive moods. The SA dimension represents symptoms of physiological hyper arousal, such as trembling, shaking, dizziness, sweating, and heart racing.

Four Dimensional Symptom Questionnaire (4DSQ) [34]: the somatization scale is one of the 4 scales of the Four-Dimensional Symptom Questionnaire, a self-report questionnaire designed to assess common psychological symptoms in primary care.

Women's Health Initiative Insomnia Rating Scale (WHI-IRS) [35]: the list consists of five questions concerning sleep in the past month. The five items address trouble falling asleep, waking up during the night, early morning awakenings, trouble getting back to sleep after waking up, and sleep quality. Although developed by the WHI, the items of this instrument are not sex-specific.

The five screening items of the Beck Scale for Suicide Ideation (BSS) [36]: the BBS is a valuable tool for clinicians to examine suicidal intent in patients. Developed for use with patients of 17 years and up, the BSS provides a good starting point for a clinician's more detailed examination of a patient's suicidal intent.

### Analyses

All statistics were performed using SPSS 15 (SPSS Inc, Chicago, Illinois, USA). The three analyses done are 1) Effect of the duration of HIV; 2) Comparison with ATHENA; and 3) Comparison with depressed homosexual men from NESDA. We used ANOVA to compare group means of continuous variables and the Pearson chi-square statistic to test for differences between groups on discrete variables. When cells had an expected count less than 5, the likelihood ratio is reported instead of the Pearson chi-square statistic.

When comparing HIV-infected and non-infected depressive homosexual patients, several covariates were entered in the statistical model. A particularly important covariate was the clinical setting. It seems likely that patients who seek help for current mental health problems show more symptoms of depression and anxiety than those who do not seek help or are in remission. While all HIV-positive patients had been referred to the mental health clinic for their mental health problems, this was only true for 42% of the HIV-negative depressed subjects. Other covariates that we adjusted for were Dutch origin, age, partner, and work status. Adjustments were done with linear regression analysis for continuous outcomes, and logistic regression for dichotomous variables.

## Results

### Cohort of patients from the outpatient clinic for HIV and Mental Health (N = 196)

Between March 2006 and September 2009, 291 new patients visited the outpatient clinic for HIV and Mental Health. 56 patients were excluded from the study because they either had no HIV-infection, could not read and write Dutch, or had not completed the diagnostic interview. Another 39 patients refused, leaving a total of 196 patients who participated in the study. Non-responders were more often of a non-Dutch origin (42% vs. 26%, p = 0.043) and were less likely to have a partner (32% vs. 49%, p = 0.051). No differences were found on other demographics or DSM-IV diagnoses.

The demographic characteristics of the HIV-infected patients seeking treatment are listed in Table 1. We could not obtain all demographic characteristics from each patient. Respondents had a mean age of 42 and the mean age at which the HIV-infection was diagnosed was 35.The majority of the patients were homosexual men (77.5%), 49% of the patients had a partner, and 60% had a paid job for more than 12 hours per week. Most patients (68%) had a history of psychiatric treatment before they came to the outpatient clinic. The majority of the patients had Dutch nationality; however, 26% were not originally from the Netherlands. In total, 29 different original nationalities were represented. The level of education was high, only 7% had only a basic education. The respondents reported heavy drugs use 30 days prior to the assessment: 26% had used cannabis and 27% ecstasy, speed or cocaine and 15% had used more than one type of drug.

**Table 1:** Demographic characteristics of the 196 patients compared with 5304 patients in Amsterdam in follow-up at the end of 2007 in the ATHENA cohort (database of the Stichting HIV Monitoring)

	**Amsterdam-Clinic**		**Amsterdam-ATHENA**		
	***N or mean***	Valid% or range *	***N or mean***	***Valid% or range***	***p value***
**Age at intake**	42 (± 8.8)	(18–65)	43.8 (± 10.6)	(1–81.9)	p = 0.03
**Age at HIV diagnosis**	35 (± 8.8)	(15–64)	35.5 (± 10.2)	(0–81.5)	p = 0.6
**Male**	172	87.7%	4380	79.6%	p = 0.06
**Homosexual men**	148	77.5%	3437	64.8%	p = 0.002
**Has a partner**	91	48.9%			
**Has a paid day job(>12 hr/week)**	112	60.2%			
**Has a psychiatric history**	128	68.4%			
**Has a Dutch origin**	143	74.1%	2886	54.4%	p < 0.0001
**Drug used past 30 days**	75	43.4%			
**Cannabis**	45	26.3%			
**Ecstasy, speed, cocaine**	46	26.9%			
**Heroin, LSD**	2	1.2%			
**Education**					
**Basic**	13	7.2%			
**Intermediate**	94	52.2%			
**High**	73	40.6%			
**Years of HIV**	7.0 (± 6.3)	(0–25)	8.3 (± 6.0)	(0–26.5)	p < 0.001
**0 – 1**	30	15.8%	436	8.3%	p < 0.001
**1 – 5**	65	34.2%	1458	27.9%	
**5 – 10**	43	22.6%	1432	27.3%	
**> 10**	52	27.4%	1910	36.5%	
**CD4 cells/mm3**	488 (± 234.7)	(20–1300)	522 (± 258)	(0–2150)	p = 0.1
**0 – 200**	11	7.0%	332	7.5%	p = 0.004
**201 – 500**	93	58.9%	2014	45.7%	
**> 500**	54	34.2%	2060	46.8%	
**Is on antiviral treatment**	132	68.8%	4119	77.7%	p = 0.004

* Due to missing values, the N varied from 158 (CD4 count) to 196 in the clinic and from 4406 (CD4 count) to 5304 in the ATHENA cohort.

Most patients (85%) had been infected with HIV for over one year. The average CD4-count was 488; 7% of the patients had fewer than 200 CD4-cells/mm^3^. We have no systematic information about opportunistic infections or HIV-related tumors. The majority of patients (68%) were on antiretroviral treatment.

### Comparison with the general HIV-infected population in Amsterdam

We found no difference between the two groups in age of HIV diagnosis; neither did we consider the difference in age of intake relevant. However, our cohort consisted of more men, especially more homosexual men and there were more patients of Dutch origin. More patients were within the first years of their infection, and fewer patients were using antiretroviral medication, but no substantial difference appeared in the mean number of CD4-cells, except for the number of patients with more than 500 CD4 cells/mm3

We obtained a DSM-IV diagnosis from 196 patients of the patients included in the study. The results are listed in Table 2. More than 50% of the patients were diagnosed with a major depression disorder, a dysthymic disorder, or a combination of both disorders. Anxiety disorders were less frequently present (21%). 10% of the population was diagnosed with drugs dependence or abuse, but only 5% with an alcohol dependence or abuse. Bipolar and psychotic disorders were not often diagnosed: in total more than 4% of the patients had one of these diagnoses. Adjustment disorders with depressive, anxious, or mixed characteristics were diagnosed in less than 10% of the population. Additional diagnoses like phase of life problem, bereavement or identity problem were determined in more than 30% of the population, usually as an additional diagnosis, sometimes as the main diagnosis.

**Table 2:** DSM-IV diagnosis

		**N**	**%**
**Depression**			
	**MDD without dysthymic disorder**	51	26.0%
	**Dysthymic disorder without MDD**	34	17.3%
	**MDD and dysthymic disorder**	18	9.2%
	**MDD or dysthymic disorder with comorbid anxiety disorder**	29	14.8%
**Anxiety disorder**		42	21.4%
**Substance use (dependence or abuse)**			
	**Alcohol without drugs**	11	5.6%
	**Drugs without alcohol**	20	10.2%
	**Alcohol and drugs**	4	2.0%
**Bipolar disorder II, hypomanic or psychotic disorder**		9	4.6%
**Adjustment disorders**		19	9.7%
**Other problems (V-codes)**		61	31.1%
	**Relational problems**	16	8.2%
	**Additional problems (such as bereavement, phase of life problem, identity problem, occupational problem)**	41	20.9%

### Effect of the duration of the HIV-infection

We divided our patients into four groups, based on the time since their HIV had been diagnosed. The effects of the duration of the HIV-infection are listed in Table 3. Patients who had been infected with HIV for more than ten years were significantly older, were less likely to have a paid job and more often had a history of psychiatric treatment. No differences were found on gender, sexual orientation or having a partner. As expected, the CD4-count first rises and then falls. When it comes to psychopathology, no significant differences were found between the four groups in terms of depressive or anxious symptoms, suicidal ideation and suicide attempts, drug use, or somatization.

**Table 3:** Effects of the duration of the HIV-infection

	***0 – 1 yr***	***1 – 5 yr***	***5 – 10 yr***	***> 10 yr***	***p-values***	***p-values***
	***(n = 30)***	***(n = 65)***	***(n = 43)***	***(n = 52)***	***(overall)***	***(0 vs.10 yrs)***
**Age**	41.0 (± 10.9)	38.9 (± 8.4)	41.6 (± 7.8)	47.1 (± 6.5)	0.000 ^*1*^	0.002 ^*1*^
**Male**	93.3%	86.2%	86.0%	86.5%	0.723 ^*3*^	0.327 ^*3*^
**Homosexual men**	83.3%	83.1%	81.4%	76.0%	0.778 ^*2*^	0.438 ^*2*^
**Dutch origin**	66.7%	70.8%	74.4%	84.6%	0.234 ^*2*^	0.059 ^*2*^
**Has a partner**	48.1%	44.4%	44.2%	60.0%	0.341 ^*2*^	0.318 ^*2*^
**Paid job (>12 hr/week)**	76.9%	69.2%	58.1%	40.8%	0.005 ^*2*^	0.003 ^*2*^
**Psychiatric history**	59.3%	63.1%	64.3%	84.0%	0.049 ^*2*^	0.016 ^*2*^
**CD4 cells/mm3**	358 (± 139.8)	470 (± 185.0)	601 (± 254.6)	493 (± 279.3)	0.001 ^*1*^	0.026 ^*1*^
**IDS (depressive symptoms)**	28.5 (± 13.28)	29.1 (± 12.33)	33.2 (± 15.08)	28.9 (± 12.50)	0.341 ^*1*^	0.910 ^*1*^
**BAI (anxiety symptoms)**	19.1 (± 12.66)	16.5 (± 10.62)	20.1 (± 12.45)	14.9 (± 10.98)	0.119 ^*1*^	0.117 ^*1*^
**4DSQ (somatization)**	44.8%	48.4%	47.6%	44.2%	0.969 ^*2*^	0.959 ^*2*^
**Drug used past 30 days**	32.0%	51.7%	12.5%	38.7%	0.276 ^*2*^	0.581 ^*2*^
**Suicide ideation**	37.9%	51.6%	50.0%	48.0%	0.762 ^*2*^	0.385 ^*2*^
**Suicide attempt (life-time)**	17.9%	29.5%	21.6%	34.0%	0.377 ^*2*^	0.131 ^*2*^

^1^ ANOVA.

^2^ Pearson chi-square.

^3^ Chi-square, likelihood ratio.

Due to missing values, the N may vary per item.

### Comparison with HIV-negative depressive homosexual men

The results of the comparison between HIV-positive and HIV-negative homosexual men with a depression or a dysthymic disorder are listed in Table 4. There were no differences between the two groups regarding age, nationality, having a partner, having a paid job or educational level.

**Table 4:** HIV-infected homosexual patients and non HIV-infected homosexual patients with a depressive or dysthymic disorder

	***HIV-infected***	***Non HIV-infected***	***p-values***	***p-values***
	***(N = 84)***	***(N = 60)***	***(not adjusted)***	(adjusted *)
**Age**	42.2 (± 8.4)	44.3 (± 11.6)	0.204 ^*1*^	0.706
**Dutch origin**	76.2%	93.3%	0.005 ^*2*^	0.214
**Has a partner**	60.5%	51.7%	0.191 ^*2*^	0.965
**Paid job (>12 hr/week)**	57.1%	61.7%	0.524 ^*2*^	0.843
**High educational level**	54.5%	51.7%	0.738 ^*2*^	0.240
				(adjusted**)
**IDS (depressive symptoms)**	33.6 (± 12.79)	25.9 (± 13.38)	0.000 ^*1*^	0.357 ^*3*^
**BAI (anxiety symptoms)**	19.3 (± 12.26)	13.4 (± 10.65)	0.002 ^*1*^	0.440 ^*3*^
**Fear Q**	28.6 (± 19.94)	27.5 (±19.37)	0.761 ^*1*^	0.210 ^*3*^
**Suicide ideation**	53.6%	20.0%	0.000 ^*2*^	0.009 ^*4*^
**Suicide attempt (life-time)**	28.6%	30.0%	0.852 ^*2*^	0.967 ^*4*^
**IRS (sleep)**	10.6 (± 5.52)	9.45 (± 5.04)	0.224 ^*1*^	0.386 ^*3*^
**MASQ: positive affect**	39.6 (± 7.93)	37.6 (± 8.09)	0.161 ^*1*^	0.248 ^*3*^
**MASQ: negative affect**	28.8 (± 8.71)	23.0 (± 7.61)	0.000 ^*1*^	0.065 ^*3*^
**MASQ: somatic arousal**	18.9 (± 7.20)	16.6 (± 6.26)	0.065 ^*1*^	0.627 ^*3*^
**4DSQ score**	15.2 (± 10.76)	12.9 (± 10.00)	0.194 ^*1*^	0.882 ^*3*^
**4DSQ: somatization**	56.6%	50.0%	0.433 ^*2*^	0.831 ^*4*^
**AUDIT score (alcohol use)**	6.36 (± 5.69)	6.33 (± 5.59)	0.977 ^*1*^	0.190 ^*3*^
**If drinker: glasses per month**	29.9 (± 36.62)	22.9 (± 31.33)	0.279 ^*1*^	0.241 ^*3*^
**If drinker: glasses per session**	2.90 (± 1.77)	1.76 (± 1.91)	0.001 ^*1*^	0.003 ^*3*^
**Drugs used past 30 days**	54.3%	21.7%	0.000 ^*2*^	0.006 ^*4*^
**Cannabis**	33.3%	15.0%	0.016 ^*2*^	0.107 ^*4*^
**Ecstasy, speed, cocaine**	30.4%	10.0%	0.004 ^*2*^	0.059 ^*4*^
**Heroin, LSD**	1.4%	1.7%	0.921 ^*2*^	0.998 ^*4*^
**NEO: neuroticism**	42.8 (± 6.84)	39.0 (± 7.81)	0.002 ^*1*^	0.055 ^*3*^
**NEO: extraversion**	33.0 (± 7.16)	33.7 (± 7.18)	0.592 ^*1*^	0.866 ^*3*^
**NEO: openness**	32.7 (± 5.28)	39.6 (± 5.68)	0.000 ^*1*^	0.000 ^*3*^
**NEO: agreeableness**	40.5 (± 5.86)	42.0 (± 6.23)	0.162 ^*1*^	0.587 ^*3*^
**NEO: conscientiousness**	34.5 (± 6.77)	40.1 (± 6.94)	0.000 ^*1*^	0.001 ^*3*^

* adjusted for clinical setting.

** adjusted for clinical setting, origin, age, partner and work status.

^1^ ANOVA.

^2^ Pearson chi-square.

^3^ Linear regression, ANOVA.

^4^ Logistic regression, Pearson chi-square.

Due to missing values, the N may vary per item.

The patients with HIV were not more depressed or anxious, measured using the IDS, BAI, MASQ positive affect, and Fear Questionnaire. The MASQ negative affect showed more symptoms like fear, anger, and guilt in the HIV-infected patients. The HIV-infected patients showed more suicide ideation, but did not report having committed more suicide attempts. There was no significant difference in alcohol use measured using the total AUDIT-score and in glasses per month. However, the HIV-infected patients drank significantly more standard glasses of alcohol per session. There was a significant difference in the amount of drugs used 30 days prior to assessment. In fact, the HIV-infected patients used more drugs in general: there was a trend for higher scores on use of ecstasy, speed, cannabis, and cocaine.

There was no difference in somatization between the two groups, when measured using the 4DSQ and MASQ and in sleep disturbance measured using the IRS. The results of the NEO showed a mixed pattern: there was a trend for higher scores on the Neuroticism-scale in the HIV-infected population. There were no differences at the Extraversion and Agreeableness scale, but the scores on the Openness scale and conscientiousness scale were significantly lower.

## Discussion

The patients of the cohort from the outpatient clinic for HIV and Mental Health differ from the general HIV-infected population in Amsterdam (ATHENA) in several respects. In our cohort, more homosexual men with the Dutch nationality were represented, and women and heterosexual men and patients with a foreign nationality were underrepresented. It is unlikely that these patients had fewer mental health problems [37]. Although homosexual men have an increased risk of psychiatric problems [38], we assume that immigrants may not arrive at the clinic as frequently due to the stigma on mental health problems. It is also possible that mental problems in that group of HIV-infected patients are not sufficiently recognized by general practitioners and internists.

At the outpatient clinic, more patients were treated early in their HIV-infection compared to the general population and therefore they used less antiretroviral medication. Although we have no official data on this subject, it is possible that part of the lower use of medication is caused by poor adherence, influenced by mental health problems [3,4]. Untreated mental health problems in HIV-infected patients can have major consequences and general practitioners and internists should be trained in recognizing mental health problems in HIV-infected patients.

As expected, depression and dysthymic disorder were the most commonly occurring diagnoses in the cohort. Mood disorders are common mental disorders and the most important reason for psychiatric referral among HIV-infected patients [39]. Depression and HIV have a reciprocal relationship: with depressive symptoms there is an increased risk of getting HIV, and with HIV there is an increased risk of depressive symptoms.

There are few studies on mental disorders in patients with prolonged HIV infection and the results are inconsistent [40]. The present study found that those patients who had been infected with HIV for more than 10 years did not have more depressive or anxious symptoms, despite the fact that 84% of these patients have a psychiatric history; neither did they report more physical symptoms. Based on our research findings, we cannot conclude that a long-term HIV infection is associated with more depressive and anxious symptoms. Patients who had been infected with HIV for more than 10 years did not show more anxious and depressive symptoms than patients earlier in their infection.

Remarkably, compared with the non-infected group of patients with a depression or dysthymic disorder, the HIV-infected homosexual men with a depression or a dysthymic disorder showed no difference in depressive symptoms or sleep disturbance. Apparently, HIV infection is not a risk factor for increased depressive symptoms among homosexual men. However, there were differences between the two groups with regard to suicidal ideation and symptoms such as guilt and anger. HIV is still a disease surrounded by taboo and stigma, which can lead to feelings of shame and anger. Although showing a higher propensity for suicidal ideation, the HIV-infected group did not report more suicide attempts in the past, but there were possibly more successful suicides.

The risk of suicide is increased in HIV-infected patients, especially among gay men [15,41]. During the research period (2006–2009), none of the patients of the cohort died of suicide. In the Netherlands, between 2005–2009, 553 HIV-infected men died, and at least 38 of them(6.9% of all deaths) died of suicide, 75% of whom were homosexual men. In the general population, in a group comparable in terms of age and sex, only 1.5% died of suicide [41]. Apparently some of these patients did not or no longer received psychiatric care, but most of these patients received care in the HIV Treatment Centers. To prevent these deaths by suicide, it is important that depressive symptoms and suicide ideation are better recognized and discussed, especially among homosexual men. There is not much evidence on this subject in women and more research is necessary on this particular topic.

Although the HIV-infected group drank significantly more units per session than the non-infected group, this difference is not clinically relevant. However, they also used substantially more drugs than the HIV-negative group (more than 50% of the HIV infected group had used drugs in the past 30 days) which is a risk factor for transmission of HIV [17-19]. It is not clear whether patients used more drugs before their HIV infection, or that drug use had mainly increased afterwards. However, to prevent HIV transmission, reducing drug use is vitally important.

Personality traits, measured using the NEO, showed a mixed pattern. In the HIV infected group, neuroticism showed higher results, while openness and conscientiousness showed lower results. High scores on neuroticism and low scores on conscientiousness are associated with HIV risk behaviours [22]. The scores on neuroticism and conscientiousness are risk factors for contracting and spreading HIV. Treatment of certain personality traits seems useful in reducing the risk of HIV risk behaviors, although further research is necessary to confirm this hypothesis [42]. High scores on openness, extraversion, and conscientiousness are associated with slower HIV progression [43]. It is unclear how low scores for openness and conscientiousness should be interpreted for HIV progression.

The present study faces some inherent limitations. We were only able to assess HIV infected patients who had sought help. We have no information on the characteristics of HIV infected patients with mental health problems who did not visit our clinic. Another limitation was the small sample size of the non-HIV-infected homosexual patients with a depressive or dysthymic disorder, resulting in low statistical power.

## Conclusion

Depression and dysthymic disorder are the most common diagnoses among HIV-infected patients who seek treatment for mental health problems. Although we found no difference in severity of depressive symptoms between HIV-infected and non HIV-infected homosexual men with a depressive or dysthymic disorder, the HIV-infected patients show more feelings of anger and guilt and experience more suicide ideation. Habitual drug use and different personality traits, such as neuroticism and lack of conscientiousness, are risk factors for contracting and spreading HIV. These risk factors are more often diagnosed in HIV-infected homosexual men with a lifetime depression or dysthymic disorder than the non-infected population. Untreated mental health problems in HIV-infected patients can have serious consequences. General practitioners and internists should be trained to recognize mental health problems in HIV-infected patients.

## Competing interests

The authors declare that they have no competing interests.

## Authors’ contributions

AS: design of the study, acquisition of data, interpretation of data, drafting and revising the manuscript, final approval. GvG: acquisition of data, analysis and interpretation of data, revising the manuscript. JHS: analysis and interpretation of data, revising the manuscript, final approval. All authors read and approved the final manuscript.

## Pre-publication history

The pre-publication history for this paper can be accessed here:

http://www.biomedcentral.com/1471-244X/13/35/prepub
